# Effect of fine needle aspiration cytology for diagnosing various spectrums of head and neck lesions

**DOI:** 10.6026/973206300210708

**Published:** 2025-04-30

**Authors:** Vandana Chandan Kumar, Chandan Kumar

**Affiliations:** 1 Department of Pathology, Nalanda Medical College and Hospital, Patna, Bihar, India; 2 Department of ENT, Nalanda Medical College and Hospital, Patna, Bihar, India

**Keywords:** Fine needle aspiration cytology, head and neck lesion, diagnosis

## Abstract

The effect of Fine Needle Aspiration Cytology (FNAC) in identifying lesions in the head and neck (H&N) area is of interest. The patients with head and neck lesions underwent both fine needle aspiration cytology and histological evaluation. Fine-needle aspiration cytology assessment identified lymph node lesions (47%) as the predominant kind, succeeded by thyroid lesions (24.64%), salivary gland lesions (10%), and bone lesions (12.21%). The diagnostic accuracy of fine-needle aspiration cytology for diverse head and neck lesions was markedly elevated, exhibiting specificity of 93.12%, sensitivity of 90.41%, negative predictive value of 91.25%, and positive predictive value of 90.26%. Fine needle aspiration cytology can serve as an effective technique for diagnosing diverse lesions in the head and neck region.

## Background:

Lesions of the jaws, thyroid gland lesions, salivary gland lesions, superficial lymph node lesions, and superficial skin and soft tissue growths are among the various types of lesions in the head and neck (H&N) region [1, 2]. There are many different types of head and neck lesions. Hence,efforts are currently being made to develop a straightforward, accurate, and dependable diagnostic method that would facilitate patient care [3, 4]. For nearly a century, diagnostic histology has been the foundation for diagnosing head and neck disorders. The use of fine needle aspiration cytology to detect head and neck lesions is growing in popularity nowadays. For several individuals, the fine-needle aspiration cytology outcome has been enough to guide decisions regarding therapy when paired with the assessment of clinical and radiologic data [5, 6]. Merely a tiny percentage of patients required a biopsy before the final treatment [7, 8]. On its own, fine-needle aspiration cytology is a reliable diagnostic technique for a wide variety of bone cancers, both malignant and benign [9, 10]. In many facilities, fine-needle aspiration cytology combined with CytoDiagnostics for bone abnormalities is currently a standard practice. Fine-needle aspiration cytology is less intrusive to the bone than an open biopsy, allows for many samples without complications and does not result in scarring. Ensuring fundamental sterility mitigates the chance of infection. Fine-needle aspiration cytology is a straightforward, expeditious, and cost-effective outpatient technique [9, 10-11]. The primary objectives for establishing a bacteriologic diagnosis include determining the morphological characteristics indicative of benignity or malignancy, investigating potential secondary bone tumors, and occasionally obtaining material samples from osteolytic lesions that are radiologically suspected to be osteomyelitis [12, 13, 14 15-16]. Rather than needing an open biopsy for a definitive histological diagnosis, numerous individuals are routinely observed over prolonged durations to see subsequent radiographic alterations [11, 12, 13, 14-15]. Occasionally, this postpones critical or hazardous diagnosis. Establishing a satisfactory correlation between clinical-radiological findings and fine-needle aspiration cytology may offer physicians a conservative option to more invasive procedures, such as open biopsy [16, 17, 18, 19- 20]. Therefore, it is of interest to determine the role and efficacy of fine-needle aspiration cytology in the diagnosis of lesions inthe head and neck region.

## Methods and Materials:

This study was an observational study at the Department of Pathology, Nalanda Medical College and Hospital, Patna, Bihar, India from January 2022 to December 2022. Results have been compiled, examined, and reviewed after data were extracted from the Department of Pathology's patient reports, which were maintained. Radiological as well as clinical information was taken from patient records. The patients with head and neck lesions underwent both fine-needle aspiration cytology as well as histopathological examination.

## Inclusion criteria and exclusion criteria:

All patients with histopathologically proven lesions in the head and neck region were isolated from other lesions that were aspirated. Crucial demographic information, along with clinical details, was recorded and tallied. Fine-needle aspiration cytology carried out under ultrasonography guidance was not included in this study.

## Methodology:

Both alcohol-fixed and air-dried smears, stained with Papanicolaou (PAP) and May-Grünwald-Giemsa (MGG) stains, respectively, as well as smears stained with head and neck stain, were examined. Where appropriate, the results were also correlated with specific stains, such as the Ziehl-Neelsen (ZN) stain. The lymph nodes, thyroid, skin, soft tissues, and salivary glands were the categories assigned to the fine-needle aspiration cytology sites.

## Statistical methods:

To estimate the frequency of different disease states, data was collated, slides were examined if needed, and percentages were computed. Where possible, the cytological diagnosis was correlated with histopathology, and the statistical tool EpiInfo was used to assess the consistency rate, sensitivity, and specificity values, with histopathology diagnosis serving as the gold standard.

## Results:

In this study, a total of 500 cases of head and neck lesions underwent fine-needle aspiration cytology. Fine-needle aspiration cytology evaluation revealed that lymph node lesions (47%) were the most common, followed by thyroid lesions (24.64%), salivary gland lesions (10%), and bony lesions (12.21%). The diagnostic accuracy of fine-needle aspiration cytology in diagnosing different head and neck lesions was significantly greater (Table 1). TB Lymphadenitis (50.21%) was the most common lymph node lesion, followed by Reactive Lymphadenitis (26%). fine-needle aspiration cytology accuracy in diagnosing various lymph node lesions was higher compared to biopsy (Table 2). Colloid goiter (70.31%) was the most common thyroid lesion, followed by follicular neoplasm (15.62%) as diagnosed by FNAC. The accuracy of fine-needle aspiration cytology as compared to biopsy was significantly higher (Table 3). Pleomorphic adenoma, a benign salivary gland tumor, was the most common type of salivary gland tumor, constituting 34.61% of all salivary gland tumors, according to fine-needle aspiration cytology. The accuracy of fine-needle aspiration cytology as compared to biopsy was significantly higher (Table 4). The epidermal cyst was the most common soft tissue lesion, as determined by fine-needle aspiration cytology. The accuracy of fine-needle aspiration cytology as compared to biopsy was significantly higher (Table 5). Odontogenic and Non-odontogenic cysts (24.13%), Benign Neoplasms (27.58%) and Malignant neoplasms (20.68%), according to fine-needle aspiration cytology (Table 6). The overall diagnostic parameters for diagnosing different jawbone lesions were significantly higher.

## Discussion:

Many patients are routinely monitored for extended periods to detect successive radiographic changes, rather than undergoing an open biopsy to establish a definitive histological diagnosis [13, 14, 15, 16, 17- 18]. This can occasionally postpone crucial or risky diagnoses. Fine-needle aspiration cytology can offer physicians a conservative alternative to more invasive procedures, such as open biopsy, provided that a satisfactory link is established between clinical and radiological outcomes [15, 16, 17, 18-19]. This study was conducted to determine the role and efficacy of fine-needle aspiration cytology in diagnosing swellings inthe head and neck region.In our study, fine-needle aspiration cytology evaluation revealed that lymph node lesions (47%) were the most common, followed by thyroid lesions (24.64%), salivary gland lesions (10%), and bony lesions (12.21%). The diagnostic accuracy of fine-needle aspiration cytology in diagnosing different head and neck lesions was significantly greater. The results align with recent studies demonstrating enhanced diagnostic accuracy of fine-needle aspiration cytology for various head and neck diseases [21, 22, 23, 24, 25- 26]. Fine-needle aspiration cytology does not leave a scar, permits the collection of many samples without complications, and is less intrusive to the bone compared to an open biopsy. The risk of infection is mitigated by upholding necessary sterility. Fine needle aspiration cytology is a direct, rapid, and cost-effective outpatient procedure. The principal objectives of establishing a bacteriologic diagnosis are to discover morphological indicators of benignity or malignancy, investigate potential secondary bone tumours, and, at times, acquire material samples from osteolytic lesions that are radiologically suspected to be osteomyelitis [21, 22, 23, 24, 25, 26, 27-28]. TB Lymphadenitis constituted 50.21% of the lymph node lesions, while Reactive Lymphadenitis accounted for 26%. The accuracy of fine-needle aspiration cytology in diagnosing diverse lymph node lesions surpassed that of biopsy. Colloid goitre (70.31%) was the predominant thyroid lesion, succeeded by follicular neoplasm (15.62%) as identified using FNAC. The precision of fine-needle aspiration cytology in comparison to biopsy was markedly superior. Our findings are corroborated by additional research demonstrating enhanced diagnostic efficacy of fine-needle aspiration cytology in head and neck lymph node lesions and thyroid gland lesions [22, 23, 24-25]. The head and neck region has numerous lesions, encompassing those of the jaws, thyroid, salivary glands, superficial lymph nodes, as well as superficial skin growths and soft tissue lesions. Numerous diverse types of head and neck injuries exist, prompting efforts to establish a simple, reliable, and consistent diagnostic method to enhance patient therapy [13- 14]. Diagnostic histology has served as the foundation for identifying head and neck issues for nearly a century. The identification of head and neck lesions via fine-needle aspiration cytology is becoming progressively prevalent. The fine-needle aspiration cytology result, when integrated with clinical and radiologic evaluations, has shown adequate for numerous patients in guiding therapeutic options [15, 16]. Pleomorphic adenoma, a benign neoplasm of the salivary glands, represented the predominant variant, accounting for 34.61% of all salivary gland tumours as determined by fine-needle aspiration cytology. The precision of fine-needle aspiration cytology in comparison to biopsy was markedly superior. The epidermal cyst was identified as the predominant soft tissue lesion using fine-needle aspiration cytology. The precision of fine-needle aspiration cytology in comparison to biopsy was markedly superior. Odontogenic and nonodontogenic cysts constitute 24.13%, benign neoplasms account for 27.58%, and malignant neoplasms represent 20.68%, as per fine-needle aspiration cytology findings. The diagnostic parameters for identifying various jawbone lesions were markedly elevated. The observations correspond with the results of other studies. These trials demonstrated considerable fine-needle aspiration cytology efficacy in salivary gland lesions, soft tissue lesions, and osseous jaw lesions. A minimal fraction of people necessitated a biopsy prior to obtaining their definitive treatment regimen [21, 22]. Fine-needle aspiration cytology is a viable diagnostic technique for a diverse array of bone malignancies, encompassing both benign and malignant forms, when utilised independently [19, 20]. Fine needle aspiration cytology in conjunction with Cyto-Diagnostics for bone anomalies is presently a regular practice in numerous medical centres [29, 30].

## Conclusion:

FNAC is an efficient method for diagnosing various lesions in the head and neck region.

## Figures and Tables

**Table 1:** Distribution of head and neck lesions (n=500 cases) according to fine-needle aspiration cytology diagnosis and diagnostic parameters about biopsy diagnosis

	**Lymph node**	**Thyroid swellings**	**Salivary Gland**	**Soft tissue & miscellaneous**	**Bony jaw lesions**
No	235	123	52	32	58
Percentage	47	24.64	10	6.15	12.21
Sensitivity (%)	93.12				
Specificity (%)	90.41				
NPV (%)	91.25				
PPV (%)	90.26				

**Table 2:** Distribution of lymph node lesions (n=235) according to fine-needle aspiration cytology diagnosis and diagnostic parameters about biopsy diagnosis

		**Inflammatory**		**Malignant**
	**Reactive Lymphadenitis**	**Acute suppurative lymphadenitis**	**TB Lymphadenitis**	**Metastatic carcinoma**
No	65	45	118	7
Percentage	26	18	50.21	2.67
Sensitivity (%)	91.04			
Specificity (%)	92.56			
NPV (%)	91.27			
PPV (%)	92.37			

**Table 3:** Distribution of various thyroid lesions (n=123) according to fine-needle aspiration cytology diagnosis and diagnostic parameters about biopsy diagnosis

	**Inflammatory**		**Benign Lesions**		**Malignant Lesions**
	**Hashimoto's thyroiditis**	**Colloid Goitre**	**Suspicious of Follicular neoplasm**	**Follicular neoplasm**	**Papillary thyroid carcinoma**
No	6	86	11	20	2
Percentage	4.68	70.31	9.37	15.62	1.56
Sensitivity (%)	90.48				
Specificity (%)	92.38				
NPV (%)	90.11				
PPV (%)	89.46				

**Table 4:** Distribution of various salivary gland lesions (n= 52) according to fine-needle aspiration cytology diagnosis and diagnostic parameters about biopsy diagnosis

		**Inflammatory**		**Benign**	
	**Acute sialadenitis**	**Chronic sialadenitis**	**Pleomorphic Adenoma**	**Warthin's tumor**	**Malignant Lesions**
No	18	10	18	6	0
Percentage	34.61	19.23	34.61	11.53	0
Sensitivity (%)	92.49				
Specificity (%)	92.75				
NPV (%)	91.28				
PPV (%)	92.27				

**Table 5:** Distribution of soft tissue and miscellaneous (n=32) according to fine-needle aspiration cytology diagnosis and diagnostic parameters about biopsy diagnosis

	**Soft tissue and Miscellaneous Lesions**	
	**Epidermal cyst**	**Lipoma**
No	13	3
Percentage	81.25	18.75
Sensitivity (%)	91.48	
Specificity (%)	92.57	
NPV (%)	93.17	
PPV (%)	91.37	

**Table 6:** Distribution of bony jaw lesions (n= 58) according to fine-needle aspiration cytology diagnosis and diagnostic parameters about biopsy diagnosis

	**Odontogenic and nonodontogenic cysts**	**Benign Neoplasms**	**Malignant neoplasms**	**Osteomyelitis**	**Fibro-osseous Lesions**	**Metastatic malignancy**
No	14	16	12	6	6	4
Percentage	24.13	27.58	20.68	10.34	10.34	6.89
Sensitivity (%)	91.12					
Specificity (%)	92.41					
NPV (%)	90.25					
PPV (%)	91.26					
